# Bacterial evolution in PCD and CF patients follows the same mutational steps

**DOI:** 10.1038/srep28732

**Published:** 2016-06-28

**Authors:** Lea M. Sommer, Mikkel Christian Alanin, Rasmus L. Marvig, Kim Gjerum Nielsen, Niels Høiby, Christian von Buchwald, Søren Molin, Helle Krogh Johansen

**Affiliations:** 1 Novo Nordisk Foundation Center for Biosustainability, Technical University of Denmark, Hørsholm, Denmark; 2 Department of Otorhinolaryngology - Head and Neck Surgery and Audiology, Copenhagen University Hospital, Rigshospitalet, Denmark; 3 Department of Clinical Microbiology, Copenhagen University Hospital, Rigshospitalet, Denmark; 4 Center for Genomic Medicine, Copenhagen University Hospital, Rigshospitalet, Denmark; 5 Danish PCD Centre, Paediatric Pulmonary Service, Department of Paediatrics and Adolescent Medicine, Copenhagen University Hospital, Rigshospitalet, Denmark; 6 Institute of Immunology and Microbiology, University of Copenhagen, Denmark; 7 Department of Systems Biology, Technical University of Denmark, Lyngby, Denmark

## Abstract

Infections with *Pseudomonas aeruginosa* increase morbidity in primary ciliary dyskinesia (PCD) and cystic fibrosis (CF) patients. Both diseases are associated with a defect of the mucociliary clearance; in PCD caused by non-functional cilia, in CF by changed mucus. Whole genome sequencing of *P. aeruginosa* isolates from CF patients has shown that persistence of clonal lineages in the airways is facilitated by genetic adaptation. It is unknown whether this also applies to *P. aeruginosa* airway infections in PCD. We compared within-host evolution of *P. aeruginosa* in PCD and CF patients. *P. aeruginosa* isolates from 12 PCD patients were whole genome sequenced and phenotypically characterised. Ten out of 12 PCD patients were infected with persisting clone types. We identified convergent evolution in eight genes, which are also important for persistent infections in CF airways: genes related to antibiotic resistance, quorum sensing, motility, type III secretion and mucoidity. We document phenotypic and genotypic parallelism in the evolution of *P. aeruginosa* across infected patients with different genetic disorders. The parallel changes and convergent adaptation and evolution may be caused by similar selective forces such as the intensive antibiotic treatment and the inflammatory response, which drive the evolutionary processes.

*Pseudomonas aeruginosa* is an opportunistic pathogen that frequently causes chronic infections in the upper and lower airways of primary ciliary dyskinesia (PCD) and cystic fibrosis (CF) patients^1^,^2^,^3^,^4^. Patients are usually colonised in childhood, but despite intensive antibiotic therapy a chronic infection is rarely prevented.

Stasis of respiratory secretions is inevitable in patients with PCD and CF predisposing both patient groups to airway infections, inflammations and declining lung function^3^,^5^. In healthy people inhaled pathogens are phagocytised or entrapped and removed by mucociliary clearance with constant beating of motile cilia. The cilia drive the mucus and pathogens out of the airways where they are cleared by coughing, expectoration, or swallowing of sputum. However, both PCD and CF are autosomal recessive disorders where the function of airway clearance is impaired: either directly in PCD by mutations resulting in structural and functional abnormalities of the cilia^6^, or indirectly in CF by mutations in a chloride ion channel resulting in dehydrated mucus in the airways, thereby impairing the ciliary movements^7^,^8^,^9^.

We hypothesise that if the main selective pressures found in the PCD and CF airways are the host immune defence system and the frequent presence of antibiotics, then the adaptation and evolution of *P. aeruginosa* should not differ significantly between the two groups of patients. However, if the main selective pressure is the immediate environment comprising the structure and composition of the mucus found in the airways, then the adaptation and evolutionary pathways could differ significantly.

Investigations of *P. aeruginosa* in CF patients have shown how the bacterium can adapt and evolve in the CF lung environment via mutational and phenotypic changes^10^,^11^,^12^,^13^,^14^,^15^,^16^,^17^. The most important characteristics include overproduction of alginate (mucoidity), slow growth, altered biofilm mode of growth, loss of motility, quorum sensing and protease production^10^,^13^,^18^,^19^,^20^,^21^. These adaptational changes are to a large degree associated with antibiotic treatments, the activity of the immune defence, and specific physico/chemical characteristics of the CF mucus. However it is not clear to what degree these factors independently and together impact the adaptation and evolution of *P. aeruginosa*. Similar adaptive and evolutionary investigations have to our knowledge never been performed in *P. aeruginosa* from PCD patients.

In order to understand the adaptation and evolution across the two different diseases of defective mucociliary clearance, we whole genome sequenced and phenotypically characterised *P. aeruginosa* isolates longitudinally from PCD airways in order to compare these with the evolutionary processes previously identified in the airways of CF patients^10^,^12^,^18^,^19^,^20^,^22^,^23^.

## Materials and Methods

### Patients

We included 12 PCD patients with chronic *P. aeruginosa* lung infection. Chronic infection was based on the frequency of *P. aeruginosa* positive airway samples in combination with elevated precipitins, as defined previously^3^. All had a definitive PCD diagnosis based on presentation of the characteristic clinical phenotype, ciliary ultrastructural defects visualized by electron microscopy, high speed video recordings showing abnormal ciliary function and/or a genetic mutation recognized to cause PCD^24^. All patients were diagnosed and treated at the Danish Pediatric Pulmonary Centre and PCD Centre, Rigshospitalet, Copenhagen. Patients are followed on a routine basis every three months, where a sputum sample is collected for bacteriological investigations, and clinical data is recorded as previously outlined^25^.

### PCD bacterial isolates

Isolation and identification of *P. aeruginosa* from PCD sputum samples was carried out as previously described, independent of the underlying condition^26^. Antimicrobial susceptibility profiles were determined for eight antibiotics: piperacillin + tazobactam, imipenem, aztreonam, ceftazidime, tobramycin, colistin, ciprofloxacin, and meropenem.

**Figure 1:**
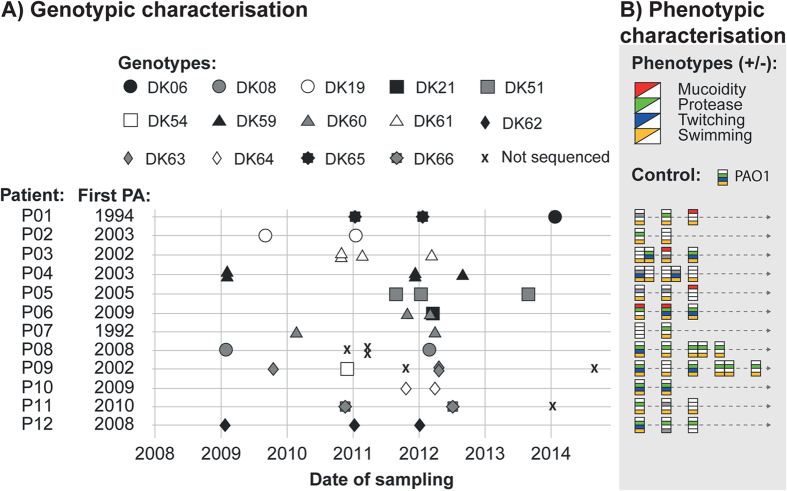
Patient and isolate overview. (**A**) Genotypic characterisation. “First PA (*Pseudomonas aeruginosa*)” shows the year the patients had the first culture of *P. aeruginosa*. Isolates denoted by “X” were not sequenced. (**B**) Phenotypic characterisation. The phenotypic characteristics are shown for 41 isolates sampled at the same time points as indicated by isolates in (**A**). When phenotypic boxes are close together isolates were sampled from the same sputum sample (se legends to Fig. 1A). A grey box indicates a non-characterisable phenotype (N/A), white indicates the absense of a phenotype, and coloured boxes indicate the presence of a phenotype.

Thirty-five longitudinal *P. aeruginosa* isolates were whole genome sequenced, on average 2.9 isolates (range 2–5) per patient with an average timespan of 2.0 years (range 0.4–3.6) (Fig. 1A).

We also phenotypically analysed 41 longitudinal isolates, on average 3.4 isolates (range 2–6 isolates) per patient with an average timespan of 2.4 years (range 0.4–4.8 years) (Fig. 1A,B).

The median age at the PCD diagnosis was 7.4 years (range 0.1–29.4 years). The median age of the patients at the time of diagnosis of chronic *P. aeruginosa* lung infection was 15.5 years (range 9–59 years), and the median age at which the first *P. aeruginosa* isolate was included in this study was 17.5 years (range 10–62 years). The median age of the patients when they had their first *P. aeruginosa* cultured was 12 years (range 4–45 years). The median time from first cultured *P. aeruginosa* isolate to the first analysed isolate was 6 years (range 1–18 years).

### Antibiotic treatment of patients

At Rigshospitalet, chronic lung infection with *P. aeruginosa* is treated in the same way in PCD and CF patients^3^,^27^. Antibiotic treatment is initiated when a sample from the lower airways is positive for a relevant pathogen, even if clinical symptoms are absent. Chronically infected patients are treated with a combination of intravenous antibiotic therapy according to susceptibility testing, either with a broad-spectrum beta-lactam or a carbapenem in combination with an aminoglycoside, colistin or ciprofloxacin, every third month. Between intravenous courses, chronically infected patients are treated with inhalation antibiotics and oral quinolones at the discretion of the treating physician.

### Genome sequencing and analysis of PCD isolates

Genomic DNA from the 35 *P. aeruginosa* strains was prepared using the Qiagen Blood & Tissue Kit and Nextera XT Sample Preparation kit. It was sequenced on an Illumina MiSeq platform generating an average of 1,695,366 150 bp paired end reads (range of 1,251,910–3,140,054) for each library to yield an estimated average genome coverage of 58.96-fold (range of 33.28–84.76-fold). See Supplementary Table S1 for information about genomic coverage depth for individual isolates. Sequences were analysed as previously described^17^.

The identification of genes with significant mutational patterns of convergent evolution was carried out using the same principles as in Marvig *et al*.^17^ in which the observed distribution of mutations were evaluated in comparison with 1,000 iterative scenarios in which mutations were randomly introduced by genetic drift. Briefly, all genes were considered separately, and dependent on their specific size genes were more or less likely to be mutated, according to the random roulette theory. It is assumed that if all mutations are randomly introduced into the genome (i.e. no selection pressure), the size of a gene will determine the number of mutations it will acquire by chance. Furthermore, genes had to be mutated in two or more clone types. However, if a gene was only mutated in two clone types, a further constraint was added: in the 1,000 iterations the gene must not have been mutated in more than 1 clone type at any time.

### Phenotypic characterisation

#### Growth rate analysis

All isolates were incubated directly from freezing stock in a 96 well plate with 100 μl Luria Bertani (LB) broth, leaving the outer wells blank. The plates were incubated at 37 °C in a microtiter plate reader (Holm & Halby, BioTek Instruments Inc., DK-2605, model: ELX808IU) with OD (600 nm) readings every 20 minutes until all isolates reached stationary phase. All isolates were assayed with a minimum of three replicates and a maximum of seven.

Motility assays were performed on soft agar plates of LB medium (twitching: 1% agar, swimming: 0.3% agar). Plates were inoculated from single colonies using sterile toothpicks, positioning the colony at the bottom of the plate (twitching) or in the middle of the agar (swimming), both were incubated for 24 hours at 37 °C. The swimming assay was carried out in 96 well microtiter plates: a positive well was indicated by a turbid well, whereas a negative was clear. All isolates were assayed with a minimum of three replicates and a maximum of four.

Secreted protease production was assayed using LB agar (1% agar) and 3% (w/vol) skimmed milk. 200 μl agar was added to each well of a 96 well plate. After drying, a hole was made in each well, where 10 μl supernatant of an overnight liquid culture (also grown in LB at 37 °C) was deposited. After 24 h, wells were read as positive if the agar had become clear and negative if opaque. All isolates were assayed with a minimum of five replicates and a maximum of eight.

Attachment assays (*in vitro* biofilm) were performed using an overnight culture grown in LB broth at 37 °C. The culture was diluted 1:100 and 150 μl was added to each well in a 96 well plate and a start OD (600 nm) was measured. The plates were incubated at 37 °C, 150 rpm for 24 h and end point OD (600 nm) was measured. The culture was removed and the plates washed three times in tab water, where after 200 μl 0.01% crystal violet was added to each well. After 20 min at room temperature (RT) the crystal violet was removed and the plates were washed three times in tab water. 200 μl 96% ethanol was added to each well and left for 20 min, 240 rpm at RT, where after OD (620 nm) was measured. The outer wells were not used and isolates were run with six replicates in each plate (and six blank controls), and this was done a minimum of three times and a maximum of seven for each isolate.

### Accession numbers

Sequence reads from all *P. aeruginosa* isolates have been deposited in the Sequence Read Archive (SRA) and can be found at: http://www.ebi.ac.uk/ena/data/view/PRJEB12111 under accession numbers: ERS1014363-ERS1014397 (Supplementary Table S1).

### Ethics

The local ethics committee at the Capital Region of Denmark Region Hovedstaden approved the use of the samples: registration number H-4-2015-FSP. All patients have given informed consent. For patients below 18 years of age, informed consent was obtained from their parents. The study was carried out in accordance with the approved guidelines and the University Hospital Rigshospitalet approved the experimental protocol.

### Statistics

All statistical analyses were carried out using the program R^28^, when multiple testing was performed a Bonferroni correction for multiple testing was used (biofilm production).

## Results

### Phylogeny of the PCD isolates

The genomic analysis identified 14 distinct clone types, of which six have previously been identified in CF patients: DK06, DK08, DK19, DK21, DK51, and DK54^17^. This suggests that there is no single clone type, which is responsible for the infections seen in either PCD or CF patients. It has previously been documented that clone types can be transmitted between CF patients^17^, and since the Copenhagen CF clinic and the Danish PCD Centre are located at close proximity in the hospital it may increase the risk of patients being exposed to the same *P. aeruginosa* clone types. This may be caused either by (1) direct patient-to-patient transmission, (2) indirect transmission via common environmental reservoirs or (3) reservoirs at the hospital^30^,^31^.

We investigated the possibility of direct patient-to-patient transmission by measuring the genetic distances between the earliest isolates sequenced from CF^17^ and PCD patients with shared clone types, (Supplementary Table S2). In all cases 68 SNPs or more separated the isolates with shared clone types, making a recent transmission between PCD and CF patients unlikely to have occurred, if it is assumed that the within-patient mutation rate of *P. aeruginosa* is around 2.6 SNPs/year, as found for CF isolates^15^.

The PCD patients included in this study have previously been shown to be infected with independent clone types assessed by Pulsed-field-gel-electrophoresis (PFGE), and 10 out of 12 patients were found to be infected by a single primary clone type, whereas two patients (P01 and P06) had a clone type switch during the study period^3^. The whole genome sequencing of the same isolates confirms these observations (Fig. 1A).

### Adaptive evolution: Pathoadaptation

*P. aeruginosa* can be isolated from many environments in water and soil and has been shown to be capable of colonizing a variety of hosts. Colonization by *P. aeruginosa* can lead to long-term infections with genetically adapted bacteria, as has been shown specifically for airway infections in CF patients. It has been suggested that genes that are repeatedly targeted by mutations are a sign of adaptive evolution, optimising the fitness of the bacteria (pathoadaptive mutations)^15^,^16^,^17^,^32^. In CF it appears that bacteria with multiple pathoadaptive mutations are more resilient to adverse conditions and more likely to persist in the host^15^.

To identify pathoadaptive mutations in *P. aeruginosa* from the PCD patients, we identified genetic variants by the comparison of the genomes of isolates within clone types. We identified mutations that had accumulated since the earliest isolate, in this case representing the most recent common ancestor (MRCA). This was only possible for clone types that were represented in the PCD collection with more than one isolate (11 out of 14 clone types), thus excluding the clone types DK06, DK21, and DK54 from this analysis.

In total we found 417 non-synonymous mutations that accumulated in the recent evolutionary history of the 11 clone types. On the basis of the number of non-synonymous mutations that accumulated in each clone type we identified genes relevant for the adaptation to the PCD airways and found genes that had been mutated in parallel between clone types. These genes were identified using an extension of a previously described method^17^. The number of non-synonymous mutations accumulated in each clonal lineage was used to estimate the expected number of mutations to be found in each individual gene, depending on the size of the gene.

**Figure 2:**
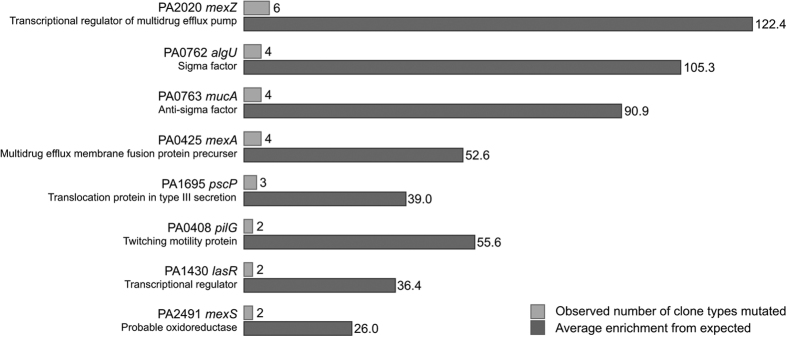
Genes mutated in more clones than would be expected by genetic drift. (**A**) Overview of the number of clones observed to be mutated in each gene, and the enrichment of genes mutated relative to expectance (p < 0.0001). (**B**) Overlap of the genes found to be important for the adaptation of *Pseudomonas aeruginosa* to the PCD airways, and genes found important for the adaptation to CF airways in three other studies: Smith (2006)^16^, Marvig (2013)^15^, and Marvig (2015)^17^. It should also be noted, that even though the genes *lasR* and *mucA* are not on the pathoadaptive gene list of Marvig (2013)^15^, they were found to have been mutated in all isolates; however, the mutations had happened in an ancestor isolate.

We identified eight genes that were more frequently mutated than would be expected to result from genetic drift; these genes were mutated in two to six clone types out of 11 (Fig. 2A, Supplementary Table S3). Our findings suggest that parallel non-synonymous mutations in these genes are the result of positive selection for mutations in genes undergoing adaptive evolution. Therefore we refer to these as candidate pathoadaptive genes in which mutations optimize pathogen fitness.

Of these eight candidate pathoadaptive genes, six have previously been identified as candidate pathoadaptive genes in the CF airways (Fig. 2B)^15^,^16^,^17^. The genes found to be repeatedly mutated in both PCD and CF are: *mucA*, *algU*, *lasR, mexZ, mexS*, and *mexA*. The two genes not identified in candidate pathoadaptive gene lists in previous CF studies are *pilG* and *pscP*. However, both genes have previously been found mutated in CF populations of *P. aeruginosa*, and although *pilG* is not identified as pathoadaptive, other pili-processing genes such as *pilQ* and *pilD* have been listed as such^17^.

It is important to note that of the eight candidate pathoadaptive genes, three are *mex* genes. In 20 out of the 35 isolates we found mutations in one or more of these *mex* genes.

The *mex* genes encode efflux pumps that have been found to be important for the resistance towards many antibiotics^33^,^34^,^35^, indicating, like in CF, that a primary selection force is the antibiotic pressure. However, antibiotic resistance was uncommon and only observed in five isolates against imipenem and one isolate against ciprofloxacin.

Mutations in *mucA* and *algU* are associated with the important hallmark of mucoidity, and have been found in CF isolates to be historically contingent^17^. This led us to investigate whether this is also the case in PCD. Six isolates were found to carry mutations in one or both genes, and in only one case did we find a mutation in *algU* without finding mutations in *mucA* (Supplementary Table S4), and in this case it was a silent mutation. The other four isolates had mutations in both genes, and only one had a mutation in *mucA* alone.

Five mucoid clones were identified among the PCD isolates. Four of these have nonsense mutations in *mucA, mucB*, or *mucD*, all of which have previously been shown to result in mucoid phenotypes^21^. Additionally, *mucA* and *mucD* mutations were identified in non-mucoid isolates in the absence of obvious second-site mutations in *algU*, which are often associated with phenotypic reversion to non-mucoidity. It has previously been shown that the reversion to a non-mucoid phenotype can be caused by second-site mutations in other genes than *algU*^21^, which is likely, also the case here.

### Adaptive evolution: Phenotypic adaptation

**Figure 3:**
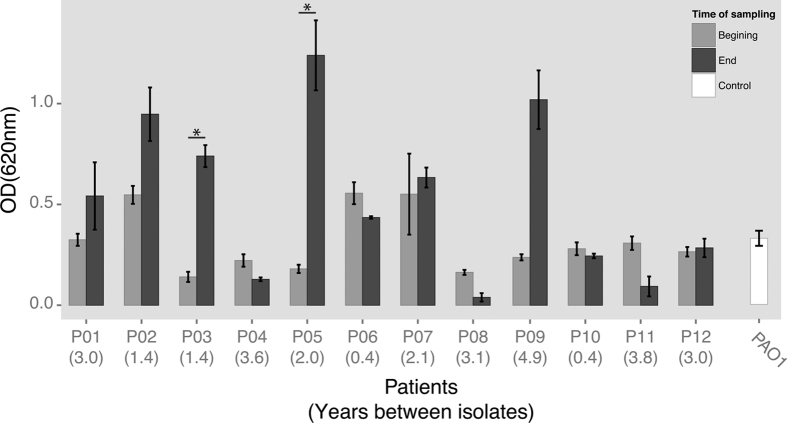
Biofilm production (attachment assay). Bar plot of the biofilm production of the first and last isolate from each patient (from Fig. 1A), including PAO1 as control, *p < 0.01 and R^2^ > 0.75 linear regression with Bonferroni correction for multiple testing. Error bars: Standard error of the mean (SEM) (SD/square root (n), n = number of samples), numbers in parenthesis below the patient IDs represent the number of years between the first and last isolate for each of the patients.

Many studies have shown that CF isolates of *P. aeruginosa* converge towards common phenotypes in relation to slow growth, loss of motility, quorum sensing, and reduced *in vitro* biofilm production of non-mucoid strains^36^. We therefore tested the phenotypic properties of the PCD isolates with regard to mucoidity, protease production (indicator of quorum sensing), swimming and twitching motility, generation time, and attachment (i.e. *in vitro* biofilm formation) (Figs 1B and 3, and Supplementary Table S5). We found that none of the longitudinal PCD isolates showed a clear pattern of becoming mucoid, and only four out of 12 patients seem to have populations that lose swimming motility and the ability to produce protease.

With regard to *in vitro* biofilm formation we found that the earliest isolates from each patient compared to the latest isolates remained equally capable of attachment and biofilm formation, only two patients’ isolates showed significant change in this trait, towards an increase in biofilm production (p < 0.01 and R^2^ > 0.8), Fig. 3. We also observed an apparent increase in biofilm formation between isolates from patient P09; nonetheless, the difference was not statistically significant. This is probably because of the large variance between measurements of the replicates of the latest isolate (0.8–1.3). If the highest value (1.3) is removed from the data we reach statistical significance of p = 0.005 (with Bonferroni correction for multiple testing), and this is the only value that can be removed to reach statistical significance of this difference in biofilm production.

Overall, the generation time did not increase and we found a median generation time of 36 min (range: 22–104 min, n = 41) similar to wild type PAO1, which we found to have a generation time of 29 min (1.82 Standard deviation) (Supplementary Table S5). Four of the patients had *P. aeruginosa* isolates that showed significant changes in generation time over the course of infection, two increased (P05 and P12) and two decreased (P06 and P07), p < 0.05, R^2^ > 0.7.

## Discussion

The PCD patients investigated in this study have been infected with *P. aeruginosa* for varying lengths of time, but 10 out of 12 patients have an infection history more comparable to the recently described cohort of young CF patients^17^ with persistent airway infections (Fig. 1) than to chronically infected older CF patients^12^.

By genome sequencing 35 and phenotypically characterising 41 *P. aeruginosa* isolates from PCD patients, we have provided a detailed insight in the bacterial evolution within the airways of PCD patients. We have found evidence of convergent molecular evolution in eight genes of *P. aeruginosa* isolates from PCD patients, and strikingly, six of these were also found to be particularly important for the adaptation in CF airways^15^,^16^,^17^. This is an investigation strategy that has been previously reviewed in Marvig *et al*.^37^, where four different pathoadaptive gene lists were compared and overlaps were identified between the studies. These overlaps were specifically related to antibiotic resistance and gene regulation, which was also the case for the six overlapping genes identified in this study. One of the pathoadaptive genes identified in our study was *lasR*, which is associated with transcriptional regulation. This was also described by Yang *et al*., who found this regulatory gene, to be mutated within the first five years of colonisation with *P. aeruginosa* in multiple CF patients^38^. In addition, the two remaining genes (*pscP* and *pilG*) were also targeted by mutations in CF isolates^17^. Thus, parallel changes in the genome sequences probably reflect similarities in the selective pressures acting on the *P. aeruginosa* populations in the airways of the two patient groups with different underlying conditions. The finding that the repeated genome changes overlap in PCD and CF isolates suggests that the environmental conditions in PCD and CF airways are highly similar. These similarities relate in particular to antibiotic therapy as well as to the immune response and impaired mucociliary clearance^6^,^7^,^8^,^9^.

The low number of pathoadaptive mutations relative to what was recently described for young CF patients most likely reflects the much lower number of genome sequenced bacterial isolates as well as the relatively short infection time with *P. aeruginosa* that we covered in the PCD patients: 474 CF isolates collected over a median of 4.8 years versus 35 PCD isolates collected over a median of 2.4 years^17^. Interestingly, the obtained results strongly suggest that, as is the case in CF patients, many clone types of *P. aeruginosa* rapidly establish persistent infections in PCD patients, and that they accumulate pathoadaptive mutations over time (Figs 1B and 2A). In a small longitudinal study in PCD patients^39^ substantial variability in the bacterial community composition was found between the patients, in contrast to the remarkable stability, which was observed for single patients over longer periods of time. This suggests that the patients keep the same clone type over time, as also shown by the persistence of clone types seen in this study.

Isolates of *P. aeruginosa* from older chronically infected CF patients often share a number of phenotypic changes such as slow growth, loss of motility and quorum sensing, increased antibiotic resistance and overexpression of alginate resulting in a mucoid phenotype on agar plates^36^. Such phenotypic changes are expected to gradually accumulate in bacterial isolates from young CF patients. In the present analysis of phenotypic changes in PCD isolates of *P. aeruginosa* we see a pattern of slow changes of the various tested phenotypes when compared with reference strain properties. The phenotypic traits of most of the PCD isolates retained many of the *P. aeruginosa* wild type traits (PAO1), such as being motile, producing proteases, maintaining and in some cases increasing the ability to attach (producing biofilm), remaining susceptible to antibiotics and retaining the growth rate (Fig. 1B and Supplementary Table S5)^10^. One reason that we only see occasional phenotypic adaptation could be due to the relatively short sampling period, indicating that the convergence towards a phenotype comparable to the isolates from chronically infected CF patients happens much later. Moreover, the occurrence of hyper-mutator isolates frequently identified in older CF patients was not observed among the PCD isolates, which is in accordance with the very low frequency identified among isolates obtained from young CF patients^17^,^40^.

All together the maintenance of wild-type phenotypic traits in the PCD isolates parallels the modest accumulation of pathoadaptive mutations. It is also obvious that in PCD airways, the bacterial populations are often heterogeneous since many different phenotypes were identified within single patients (Fig. 1B). Furthermore, it has been shown that PCD patients sometimes clear a chronic *P. aeruginosa* infection, as a consequence of antibiotic treatment^3^. This resembles the situation in young CF patients where long-term persistent infections in some cases may be cleared, but it is in contrast to chronically infected CF patients, where clearance is rarely achieved^1^,^10^,^15^,^17^. Clearance may favour re-colonisation from the sinuses^4^ or wild type environmental isolates similar to what was observed in young CF patients^17^.

Our results show that *P. aeruginosa* can establish persistent infections in both CF and PCD patients, with the *P. aeruginosa* population gradually adapting to the airways through various mutations in specific genes. There may be differences between the CF and PCD infections with respect to the speed of adaptation, but in general it seems that the adaptive processes in the bacterial populations are very similar. This conclusion may support a therapeutic strategy for the PCD patients, which is very similar to the treatment of airway infections in young CF patients where 3-week antibiotic courses are initiated when *P. aeruginosa* is cultured from the airways^27^.

## Additional Information

**How to cite this article**: Sommer, L. M. *et al*. Bacterial evolution in PCD and CF patients follows the same mutational steps. *Sci. Rep*. **6**, 28732; doi: 10.1038/srep28732 (2016).

## Supplementary Material

Supplementary Information

Supplementary Table S1

Supplementary Table S2

Supplementary Table S3

Supplementary Table S4

Supplementary Table S5
