# Supplementary material for: Bacterial evolution in PCD and CF patients follows the same mutational steps
Source: Sci Rep. 2016 Jun 28;6:28732. doi: 10.1038/srep28732 (PMC4923847; doi:10.1038/srep28732)
Supplement: Supplementary Information [file srep28732-s1.doc]

Title

Bacterial evolution in PCD and CF patients follows the same mutational steps

Lea Mette Madsen Sommer^1^*, Mikkel Christian Alanin^2^*, Rasmus Lykke Marvig^4,7^, Kim Gjerum Nielsen**^3^**, Niels Høiby^4,5^, Christian von Buchwald^2^, Søren Molin**^1,6^**, Helle Krogh Johansen^1,4,**^

^1^Novo Nordisk Foundation Center for Biosustainability, Technical University of Denmark, Hørsholm, Denmark. ^2^Department of Otorhinolaryngology - Head and Neck Surgery and Audiology, Copenhagen University Hospital, Rigshospitalet, Denmark ^3^Danish PCD Centre, Paediatric Pulmonary Service, Department of Paediatrics and Adolescent Medicine, Copenhagen University Hospital, Rigshospitalet, Denmark **^4^**Department of Clinical Microbiology, Copenhagen University Hospital, Rigshospitalet, Denmark **^5^**Institute of Immunology and Microbiology, University of Copenhagen, Denmark ^6^Department of Systems Biology, Technical University of Denmark, Lyngby, Denmark. ^7^Center for Genomic Medicine, Copenhagen University Hospital, Rigshospitalet, Denmark.

* These two authors contributed equally to the study. Shared first authorship

**Corresponding author:

Helle Krogh Johansen, MD, DMSc

Department of Clinical Microbiology

Rigshospitalet afsnit 9301

Juliane Maries Vej 22

DK-2100 Copenhagen Ø

Denmark

Mail: [hkj@biosustain.dtu.dk](mailto:hkj@biosustain.dtu.dk),

Phone: +45 3122 8406

Fax: +45 3545 6412

**Supplementary material**

**Supplementary Table S1:** Information on sampling date, patient origin, number of reads, average coverage of the PAO1 genome, and accession numbers of sequences.

**Supplementary Table S2:** Distance (number of SNPs) between initial isolates of shared clone types, from both PCD and CF patients.

**Supplementary Table S3:** Genes of PAO1, the number of clones observed to be mutated and expected number of clones mutated, for significantly mutated genes.

**Supplementary Table S4:** Historic contingencies of mutations in *mucA* and *algU*. 1 = mutation found in gene, if not insertion or deletion the mutation is written in parenthesis, missense, nonsense, and silent. *=also contains a silent SNP.

**Supplementary Table S5:** Information on sampling date, origin, and phenotypic traits (average) of all isolates.
